# Higher Plasma Kynurenine to Tryptophan Correlates with an Increased Incidence of Mild Cognitive Impairment in Treated Metabolic Syndrome Patients

**DOI:** 10.1021/acsomega.5c09713

**Published:** 2025-12-11

**Authors:** Narumol Jariyasopit, Tiwat Phochmak, Siriphan Manocheewa, Kwanjeera Wanichthanarak, Suphitcha Limjiasahapong, Nichapa Kleebkomut, Yongyut Sirivatanauksorn, Vorapan Sirivatanauksorn, Arintaya Phrommintikul, Nipon Chattipakorn, Siriporn Chattipakorn, Sakda Khoomrung

**Affiliations:** † Siriraj Center of Research Excellence in Metabolomics and Systems Biology, 65106 Faculty of Medicine Siriraj Hospital, Mahidol University, Bangkok 10700, Thailand; ‡ Siriraj Metabolomics and Phenomics Center, 65106 Faculty of Medicine Siriraj Hospital, Mahidol University, Bangkok 10700, Thailand; § Thailand Metabolomics Association, Bangkok 10700, Thailand; ∥ Department of Biochemistry, 65106 Faculty of Medicine Siriraj Hospital, Mahidol University, Bangkok 10700, Thailand; ⊥ Cardiac Electrophysiology Research and Training Center, Faculty of Medicine, 26682 Chiang Mai University, Chiang Mai 50200, Thailand; # Center of Excellence in Cardiac Electrophysiology Research, Faculty of Medicine, 26682 Chiang Mai University, Chiang Mai 50200, Thailand; ∇ Department of Internal Medicine, Faculty of Medicine, 26682 Chiang Mai University, Chiang Mai 50200, Thailand; ○ Department of Oral Biology and Diagnostic Sciences, Faculty of Dentistry, 26682 Chiang Mai University, Chiang Mai 50200, Thailand; ◆ Center of Excellence for Innovation in Chemistry (PERCH−CIC), Faculty of Science, Mahidol University, Bangkok 10700, Thailand

## Abstract

An increase in cognitive impairment was observed in metabolic syndrome (MetS) patients. Although alterations in metabolomic profiles have been identified as potential plasma/serum biomarkers of mild cognitive impairment (MCI) and MetS, findings remain inconsistent—probably due to the heterogeneity among MetS patients and the lack of subsequent validation using targeted analysis after the initial untargeted analysis. In this study, we validated mass spectrometry-based quantitation methods and quantified amino acids, fatty acids, and tryptophan metabolites in the kynurenine pathway in the plasma of 95 treated MetS patients with and without MCI assessed by the Montreal Cognitive Assessment. We found that MCI was positively associated with the kynurenine-to-tryptophan ratio (KTR) after the adjustment for age, gender, and BMI, as well as negatively associated with C20:3 [all-Z-8,11,14] and lysine. A one-unit increase in KTR resulted in an increased probability of developing MCI by 371%. In contrast, one-unit increases in C20:3 and lysine were associated with decreased odds of developing MCI by 81 and 78%, respectively. Our finding underscores prominent neuroinflammation, beyond normal aging, in MetS patients, even under ongoing clinical treatment. It also points to the potential of KTR as a risk marker for MCI, offering a valuable complement to the existing cognitive assessments that may be influenced by the educational background. In addition, the validated metabolite data serve as an invaluable resource for future research. They can facilitate comparisons across different studies, contribute to large-scale analyses, and be used in machine learning models for discovering and validating new biomarkers.

## Graphical abstract

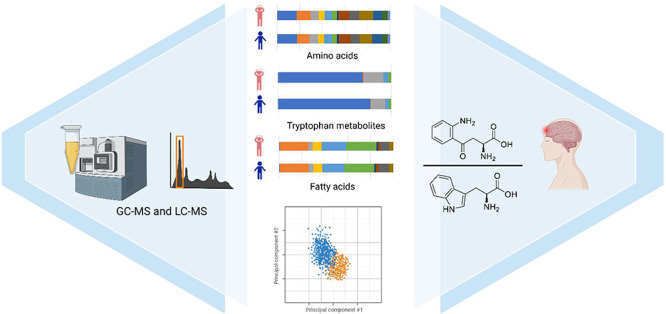

## Introduction

Metabolic syndrome (MetS) is characterized by a cluster of metabolic symptoms, including obesity, hypertension, dyslipidemia, hyperglycemia, and insulin resistance.^1^ Patients with MetS have an increased risk factor for cardiovascular, cerebrovascular diseases, and cognitive impairment.^2^ Several studies demonstrated the associations between cognitive performance and MetS.^3^ Initial clinical assessment of cognitive impairment can be assessed by cognitive screening tests such as the Mini-Mental State Examination (MMSE) and the Montreal Cognitive Assessment (MoCA).^4^ Further assessments such as neuroimaging or cerebrospinal fluid analysis will help characterize the pathology of the syndromes.^5^ While the MMSE and MoCA results can be affected by educational bias, neuroimaging and cerebrospinal fluid collection are costly or invasive. In addition, it is likely that comprehensive clinical assessments are conducted following cognitive complaints or clinical syndromes, which is when the disease may have progressed beyond the early stage. While it remains a significant challenge, early detection of the disease, using biological indicators at the preclinical stages, will offer the potential for more effective therapeutic success or delay the progression.

In the past decade, metabolomics has been widely applied to search for plasma/serum biomarkers of MCI in the general population,^6–15^ type 2 diabetic patients (T2D),^16–18^ and, to a lesser extent, MetS patients.^19^ However, the results have been inconsistent, likely due to the heterogeneity among MetS patients and the lack of subsequent validation using targeted analysis after the initial untargeted analysis. Among previously identified metabolites, tryptophan metabolites in the kynurenine pathway and branched-chain amino acids are of interest due to accumulating evidence showing a close link to insulin resistance caused by chronic inflammation.^20–23^ Fatty acids are another class of compounds that have exhibited both positive and negative contributions to cognitive functions, depending on the types of fatty acids.^6,18,24–26^ Generally, plasma/serum fatty acids are often lower in MCI and Alzheimer’s disease (AD) patients compared with cognitively unimpaired subjects,^26^ particularly docosahexaenoic acid (DHA), which has been consistently noted.^24^ On the other hand, saturated fatty acids can contribute to a pathological component of impaired cognitive function via the crossing of free fatty acids to the brain, which can initiate neuroinflammation.^27^

In this study, we determined absolute plasma concentrations of metabolites in treated MetS patients with and without MCI assessed by the MoCA. The cohort was a subpopulation of the Cohort Of patients at a high Risk of Cardiovascular Events (CORE)-Thailand registry. We targeted three classes including tryptophan metabolites in the kynurenine pathway, amino acids, and total fatty acids using gas chromatography coupled to a time-of-flight mass spectrometer (GC-TOFMS) and liquid chromatography coupled to a triple quadrupole MS (LC-TQMS). Prior to the quantitative analyses, we validated the analytical methods for accuracy and precision using the NIST Standard Reference Material 1950 Metabolites (SRM1950) in frozen human plasma. The combined metabolite data set was explored in conjunction with clinical data to investigate their associations with MCI in MetS patients.

## Materials and Methods

### Study Population

The study protocol was approved by the Institutional Ethics Committee of the Faculty of Medicine, Chiang Mai University, Chiang Mai, Thailand (Research ID: 8948, Study Code: MED-2565-08948). Written informed consents were obtained from all patients (*N* = 111) prior to the study. All of the methods were performed in accordance with the relevant guidelines and regulations. This study is a substudy of MetS patients in the Cohort Of patients at a high Risk for Cardiovascular Events (CORE)-Thailand registry, which is an ongoing prospective cohort of Thai patients with a high atherosclerotic risk. Patients were recruited from an outpatient clinic at Maharaj Nakorn Chiang Mai Hospital during the period between April 2011 and March 2014. The inclusion and exclusion criteria are provided in Figure 1.^28^ Ninety-five treated MetS patients were a subpopulation of our CORE study. All patients were divided into two groups: (1) MetS patients with MCI (MetS-MCI, *N* = 70) and (2) MetS patients with normal cognitive function (*N* = 25). MCI was determined when the MoCA score was less than 23.

**Figure 1:**
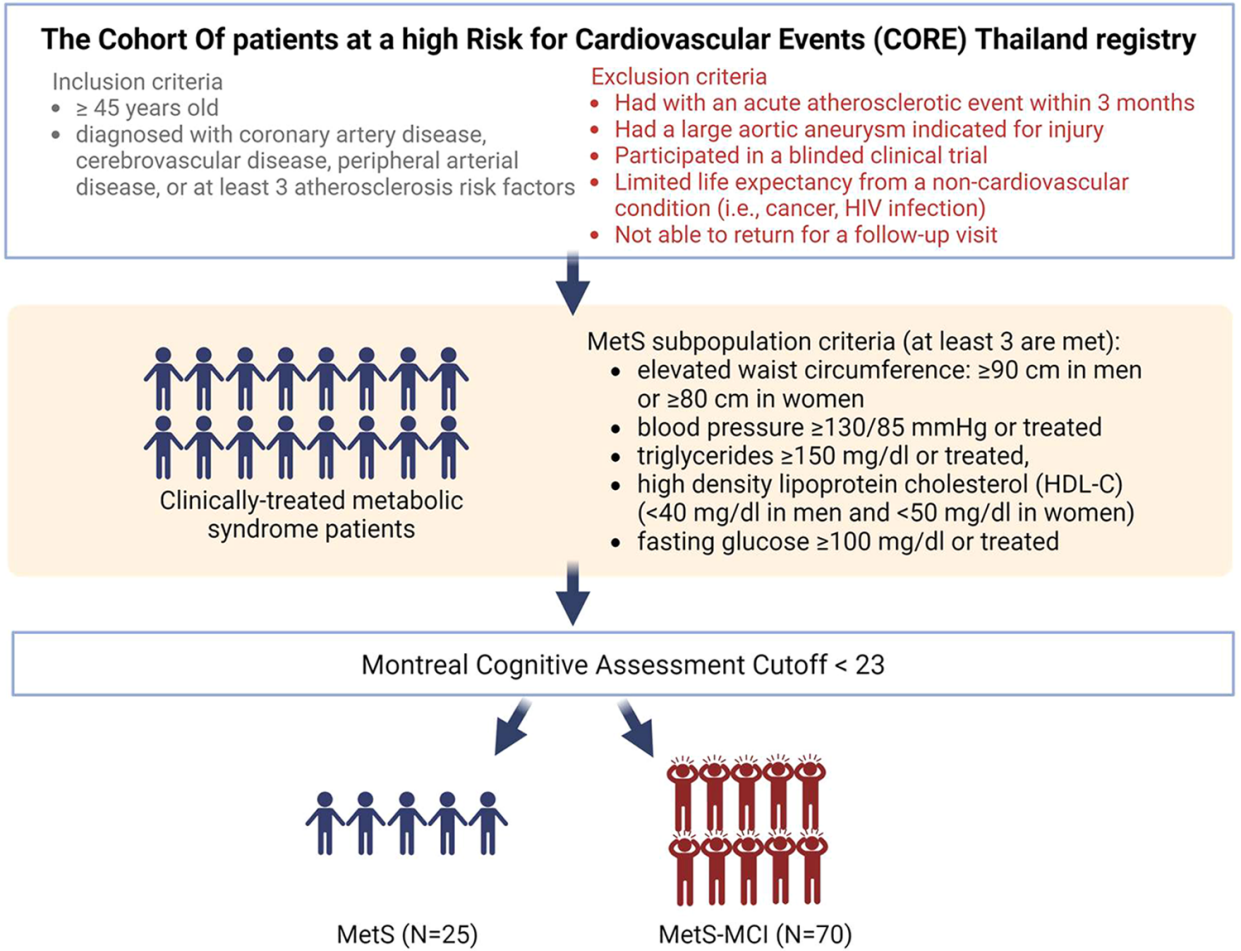
Study design.

### Chemicals

Authentic standards of amino acids, fatty acid methyl esters (FAMEs), and tryptophan metabolites in the kynurenine pathway were purchased from Sigma-Aldrich (St. Louis, MO) and Restek (Bellefonte, PA). A list of the compounds targeted in this study is provided in Table S1. Isotopically labeled internal standards, including alanine-*d*_3_, phenylalanine-*d*_5_, anthranilic acid-^13^C_6_, and picolinic acid-*d*_4_, were purchased from Cambridge Isotope Laboratories (Massachusetts, USA), and methyl nonanoate and methyl nonadecanoate were purchased from Sigma-Aldrich. NIST SRM 1950 Metabolites in frozen human plasma was purchased from Sigma-Aldrich.

### Metabolomic Analyses

Analytical methods for amino acids, total fatty acids, and tryptophan metabolites in the kynurenine pathway are summarized in Table 1.

**Table 1:** Summary of Analytical Methods Used for Amino Acids, Fatty Acids, and Tryptophan Metabolites

analysis	sample preparation	derivatizing agent	analytical platform	instrumental conditions
amino acids	• 30 μL plasma extracted with 1 mL precooled acetonitrile/isopropanol/water (3:3:2, v/v/v)	MTBSTFA + 1% TBDMSCl	GC-TOFMS	• column: Rxi-5sil MS (30 m × 0.25 mm I.D., 0.25 μm)
	• vortexed at 2,000 rpm for 2 min, stored at −20 °C for 1 h			• injector: 250 °C, splitless mode, 1 μL injection
	• centrifuged (10 min, 4 °C, 19,600×*g*)			• oven program: 50 °C (2 min) → 320 °C at 20 °C/min (3 min hold)
	• 450 μL supernatant evaporated to dryness			• transfer line/ion source: 320 °C/250 °C
	• resuspended in acetonitrile/water (50:50, v/v), centrifuged (14,000×*g*, 2 min, RT), and dried again			
	• dried sample derivatized with 50 μL MTBSTFA + 1% TBDMSCl and 50 μL acetonitrile, incubated at 100 °C, 4 h			
total fatty acids (as FAMEs)	• 50 μL plasma mixed with 0.5 mL 14% borontrifluoride in methanol	14% borontrifluoride in methanol	GC-TOFMS	• column: DB-FastFAME (30 m × 0.25 mm I.D., 0.25 μm)
	• heated at 100 °C for 1 h, cooled, then extracted with 1 mL *n*-hexane and 1 mL water			• injector: 200 °C, 1 μL injection, split 40:1
	• centrifuged 15 min, 1847×*g*, 20 °C			• oven program: 40 °C (2 min) → 150 °C (20 °C/min, 2 min) → 180 °C (10 °C/min, 3 min) → 190 °C (5 °C/min, 2 min) → 210 °C (5 °C/min) → 240 °C (15 °C/min, 3 min)
	• supernatant dried under N_2_			• transfer line/source: 250 °C/250 °C
	• reconstituted in 500 μL hexane			
tryptophan metabolites (kynurenine pathway)	• 50 μL plasma mixed with 200 μL methanol		UPLC-MS/MS	• column: HSS T3 (2.1 × 100 mm, 1.8 μm), 30 °C
	• vortexed, sonicated (10 min, RT), stored overnight at −20 °C			• injector: 5 μL injection
	• centrifuged (15 min, 13,000 rpm, 4 °C)			• mobile phase: (A) 0.1% formic acid in H_2_O; (B) 0.1% formic acid in acetonitrile
	• supernatant dried, resuspended in 100 μL water with 0.1% formic acid, sonicated (10 min, RT), centrifuged again			• gradient: 99% A → 70% A (7 min) → 30% A (at 9 min) → return to initial at 10 min (4 min hold)
				• flow rate: 0.3 mL/min
				• MRM; positive mode; capillary 1.5 kV; source 150 °C; desolvation 900 L/h at 550 °C; cone 150 L/h; nebulizer 7.0 bar; collision 0.25 mL/min

### Amino Acid Analysis

Sample extraction of amino acids from plasma was modified from a published method.^29^ In brief, 30 μL of plasma was extracted with 1 mL of precooled mixture of acetonitrile/isopropanol/water (3:3:2, v/v/v), containing isotopically labeled internal standards that included alanine-*d*_3_ and phenylalanine-*d*_5_ with final concentrations of ∼5 ng/μL. The extracts were shaken at 2,000 rpm for 2 min using a vortex mixture (Scientific Industries) before being kept at −20 °C for 1 h. The extracts were then centrifuged for 10 min at 4 °C and 19,600×*g*. A 450-μL aliquot of supernatant was evaporated to dryness using a vacuum concentrator (Labconco, MO, USA). The dried aliquot was resuspended in 450 μL acetonitrile/water (50:50, v/v) followed by centrifugation at 14,000×*g* and room temperature for 2 min. The supernatant was transferred to a new Eppendorf tube and dried under vacuum. The dried samples were derivatized with 50 μL of *N*-*tert*-butyldimethylsilyl-*N*-methyltrifluoroacetamide with 1% *tert*-butyldimethylchlorosilane (MTBSTFA + 1% TBDMSCl: *t*-BDMS) and 50 μL of acetonitrile, followed by incubation at 100 °C for 4 h.

The quantification of amino acids was carried out using a GC-TOFMS (Pegasus HRT+4D, Leco Corp., St. Joseph, MI). The separation was achieved on a nonpolar Rxi-5sil MS column (5% diphenyl-methyl polysiloxane and 95% dimethylpolysiloxane, 30 m × 0.25 mm I.D., 0.25 μM film thickness, Restek). The injection volume was 1 μL, with an injector temperature of 250 °C. The samples were analyzed in the splitless mode. Helium was used as a carrier gas at a constant flow rate of 1 mL min^–1^. The GC oven temperature program started at 50 °C (2 min hold) and ramped to 320 °C at 20 °C min^–1^ (3 min hold). Transfer line and electron impact (EI) ion source temperatures were kept at 320 and 250 °C, respectively. The MS data were acquired using ChromaTOF software (version 5.55.50, Leco Corp.).

### Total Fatty Acid Analysis

The sample preparation method was modified from a previously published protocol.^30^ To convert fatty acids to FAMEs, a 50-μL aliquot of a plasma sample was mixed in a Pyrex tube with 0.5 mL of 14% borontrifluoride in methanol and nonadecanoate (final concentration of 20 ng μL^–1^), which was used as a surrogate. The sample mixture was heated to 100 °C for 1 h. After heating, the extract was allowed to cool to room temperature before adding 1 mL of *n*-hexane and brief vortexing. One milliliter of Milli-Q water was then added to the extract, followed by vortexing for 20 s. The extract was centrifuged at 1847*g*, 20 °C for 15 min. The supernatant was dried under a N_2_ stream and reconstituted in 500 μL of hexane containing nonanoate (final concentration of 20 ng μL^–1^), used as an internal standard.

Determination of FAMEs was carried out using GC-TOFMS (Pegasus-BT, Leco Corp., St. Joseph, MI). Target FAMEs were separated on a DB-FastFAME (30 m × 0.25 mm I.D., 0.25 μm film thickness, Agilent Technologies, USA). The injection volume was 1 μL. The samples were analyzed in split mode using a split ratio of 40:1. The injector temperature was kept at 200 °C. Helium was used as a carrier gas at a constant flow rate of 1 mL min^–1^. The GC oven temperature program started at 40 °C (2 min hold), increased to 150 °C at 20 °C min^–1^ (2 min hold), increased to 180 °C at 10 °C min^–1^ (3 min hold), increased to 190 °C at 5 °C min^–1^ (2 min hold), increased to 210 °C at 5 °C min^–1^, and increased to 240 °C at 15 °C min^–1^ (3 min hold). Transfer line and EI ion source temperatures were both kept at 250 °C. The MS data were acquired by using ChromaTOF software (version 5.55.50, LECO Corp.).

### Analysis of Tryptophan Metabolites in the Kynurenine Pathway

Tryptophan metabolites were quantified using a previously published method with minor modifications.^31^ In brief, 50 μL of plasma was mixed with 200 μL of methanol containing 100 ng of picolinic acid-*d*_4_, which was used as an internal standard. After a brief vortex, the sample mixture was sonicated for 10 min at room temperature and stored at −20 °C overnight. The sample was centrifuged at 13,000 rpm at 4 °C for 15 min. The supernatant was transferred to a new Eppendorf tube and evaporated to dryness using a vacuum concentrator (Labconco, MO, USA). The sample was resuspended in 100 μL Milli-Q water with 0.1% formic acid, followed by a brief vortex and 10 min sonication at room temperature. After centrifugation at 13,000 rpm, 4 °C for 15 min, the supernatant was transferred to an LC vial.

The analysis was carried out using a Waters Acquity I-Class UPLC coupled with a Xevo TQ-Absolute MS/MS, with an electrospray ionization source. The target tryptophan metabolites were separated on an HSS T3 column, 2.1 × 100 mm, 1.8 μm (Waters, Milford, MA, USA). The column was kept at 30 °C, and the flow rate was 0.3 mL/min throughout the analysis. Mobile phases were (A) 0.1% formic acid in Milli-Q water and (B) 0.1% formic acid in acetonitrile, using a gradient program that started at 99% A, decreased to 70% over 7 min, then decreased to 30% at 9 min, returned to the initial condition at 10 min, and held for 4 min. The MS was operated in the positive multiple reaction monitoring mode to collect *m*/*z* values as given in the previous study.^31^ The optimal MS parameters were as follows: capillary voltage 1.5 kV, source temperature 150 °C, desolvation gas (N_2_) flow 900 L/h at 550 °C, cone gas flow 150 L/h, nebulizer gas (Ar) 7.0 bar, and collision gas flow 0.25 mL/min. The injection volume was 5 μL. LC-TQMS data was processed using MassLynx (Version 4.2, Waters). The quantification was performed using the matrix-matched calibration method. All of the calibration curves yielded *R* ^2^ greater than 0.99.

### Quality Assurance, Quality Control, and Metabolite Identification

Pooled samples were prepared by combining 10 μL of each individual sample. Blank samples were prepared for every sample batch by using Milli-Q water as a surrogate. For every analytical batch, a pooled sample, a blank sample, and a calibration standard or SRM1950 extract were analyzed along with the plasma samples. According to the levels of metabolite identification confidence defined by the Metabolomics Standards Initiative, all the measured metabolites were confidently identified compounds or level 1.^32^

Amino acid and fatty acid calibration solutions were prepared in neat solvents due to minimal matrix effects. However, tryptophan metabolite calibration solutions were prepared using the matrix-matched approach to account for matrix effects.^33^ No significant amounts of analytes were detected in the blank samples. Peaks were quantified only when the signal-to-noise ratio exceeded 10.^34^ Metabolite concentrations were recovery corrected. Linear ranges, limit of detection (LOD), and limit of quantification (LOQ) values are provided in Table S2. Mean percent recoveries (±standard deviations) of internal standards in the plasma samples for tryptophan metabolite, total fatty acid, and amino acid analyses were 107% (±10%, *N* = 106), 107% (±24, *N* = 106), and 94% (±39%, *N* = 106), respectively.

### Data Process and Data Analysis

Clinical parameters and metabolite concentrations were presented as medians (and IQR). Statistical differences between clinical parameters (age, years of education, and adjusted MoCA score) and metabolite concentrations were tested by the Mann–Whitney *U* test. Concentrations below LOQs were replaced by LOQs before data analysis. Metabolites with missing values greater than 30% of the total number of samples in each group were removed from the data set. The missing values were imputed by median concentrations of the metabolites within each group, followed by log2 transformation. Mann–Whitney *U* test and Spearman’s correlation analyses of the combined data set were carried out using SPSS software version 18. The multivariate analysis was performed using the Metabox2 R package.^35^ The odds ratios were calculated from binary regression models using the R package version 4.4.3. Reported *p*-values were not adjusted for multiple comparisons. Given the limited number of statistical tests performed, unadjusted *p*-values were reported to maintain interpretability and to avoid an unnecessary loss of statistical power.

## Results

### Clinical Characteristics of the Cohort

Study design is summarized in Figure 1. Demographic and clinical data for the cohort are listed in Table 2. The cohort consisted of 39 male and 56 female MetS patients who had been clinically treated. The median age of all patients is 63 (60–69) years. All patients were assessed for cognitive performance using the MMSE and MoCA tests. The MMSE scores of all patients were within the normal range. Regarding the MoCA score, patients with a MoCA score greater than or equal to 23 ^36^ were categorized as MetS patients with normal cognitive function (MetS, *N* = 25). MetS patients with a MoCA score less than 23 were categorized as MCI (MetS-MCI, *N* = 70). The significant difference in MoCA scores between the two groups (MetS: 25 (24–26) vs MetS-MCI: 19 (18–21)) was observed. The MoCA scores had been adjusted with the level of education. Median ages for MetS (60 (57–62) years old) and MetS-MCI (65 (61–71) years old) were statistically different (*p*-value <0.05). Median number of years of education for MetS-MCI (4 (4–12) years) was statistically lower than that for MetS (12 (9–16) years, *p*-value <0.001).

**Table 2:** Clinical Characteristics of the Cohort^a^

	MetS (*N* = 25)		MetS-MCI (*N* = 70)	
%female	57.5 (*N* = 14)		60.8 (*N* = 42)	
	median	IQR	median	IQR
age (years)*	60	57–62	65	61–71
weight (kg)	69.0	59.9–75.1	65.5	55.1–74.0
BMI	27.5	23.9–32.0	26.1	22.8–30.6
years of education***	12	9–16	4	4–12
glucose (mg/dL)	115	96–135	125	104.3–151.5
insulin (μU/mL)	7.1	4.7–8.1	5.1	3.0–8.4
FGF21 (pg/mL)	216.6	174.3–351.1	261.8	182.2–406.3
triglyceride (mg/dL)	111.0	73.0–173.0	115.5	86.0–141.0
total cholesterol (mg/dL)	142.0	129–179.0	147.5	126.0–192.3
HDL (mg/dL)	46.5	37.0–62.0	49.0	40.0–60.8
LDL (mg/dL)	84.5	72.0–107.0	83.0	69.0–118.5
VLDL (mg/dL)	23.0	17.0–35.0	23.5	19.3–27.0
HbA1C	7.0	5.8–7.5	7.2	6.4–8.3
adjusted MOCA score***	25	24–26	19	18–21

a * *p*-value <0.05, ** *p*-value <0.01, and *** *p*-value <0.001 (Mann–Whitney *U* test).

### Validation of the Quantification Methods Using Plasma SRM 1950

We ensured the accuracy of the quantification methods using the NIST plasma SRM 1950. Of the 12 certified amino acid concentrations, our method accurately quantified 8 amino acids, including glycine, leucine, isoleucine, lysine, proline, threonine, tyrosine, and valine, yielding percent errors of less than 30 (Table S3). It should be noted that the *t*-BDMS derivatization converts arginine to ornithine,^37^ therefore, we reported arginine as ornithine using a calibration curve derived from arginine standard. For fatty acids, eight fatty acid concentrations yielded percent errors of less than 30: C12:0, C16:0, C16:1 [Z-9], C18:0, C18:3 [all-Z-9,12,15], C18:1 [Z-9], C18:2 [Z,Z-9,12], and C22:0, with (Table S3). Because certified concentrations of tryptophan metabolites are not available in the SRM, we validated the method using spike recovery method and obtained an average %recovery (±SD) of 96.7 (±5.5). Method precision was evaluated by injecting a midrange concentration standard (8 ng/μL) three times. The average percent precision for most of the analytes was 7% (±5%), whereas histidine exhibited a higher variability of 35% (±4%).

### Quantitative Metabolomics of MetS and MetS-MCI

Median concentrations (and IQRs) of all targeted metabolites and metabolite compositions are listed in Table 3 and Figure 2, respectively. While the amino acid compositions of the two groups were quite similar (Figure 2A), the tryptophan metabolite composition of MetS-MCI showed a higher proportion of kynurenine and a lower proportion of tryptophan compared to MetS (Figure 2B). Median kynurenine concentrations in MetS and MetS-MCI were 88.95 (85.39–98.46) and 104.08 (87.34–143.94) nmol/dL, respectively, while median tryptophan concentrations in MetS and MetS-MCI were 3.36 (2.66–3.58) and 2.84 (2.51–3.36) μmol/dL, respectively (Table 3). Fatty acid composition of Met-MCI showed a slightly larger proportion of oleic acid (C18:1 [Z-9]) and a lower proportion of arachidonic acid (C20:4 [all-Z-5,8,11,14]) than that of MetS (Figure 2C). However, the median concentrations of those two fatty acids were not significantly different (Table 3). Using the Mann–Whitney *U* test, four metabolites showing significant differences between MetS and MetS-MCI were lysine, kynurenic acid, kynurenine, and eicosatrienoic acid (C20:3 [all-Z-8,11,14]) as well as the kynurenine-to-tryptophan ratio (KTR). As shown in Figure 2D, MetS had lower kynurenic acid, kynurenine, and KTR levels, but higher lysine and C20:3 [all-Z-8,11,14] concentrations compared with MetS-MCI. For MetS, median kynurenic acid, lysine, C20:3 [all-Z-8,11,14] concentrations, and KTR values were 2.64 (2.01–3.60) nmol/dL, 26.25 (21.00–30.65) μmol/dL, 23.77 (23.19–29.10) μmol/dL, and 0.0274 (0.0253–0.0326), respectively, while they were 3.49 (2.64–4.76) nmol/dL, 22.34 (19.95–26.05) μmol/dL, 20.85 (18.79–23.67) μmol/dL, and 0.0374 (0.0299–0.0492), respectively, in MetS-MCI.

**Table 3:** Median and IQR Concentrations of Amino Acids, Tryptophan Metabolites in the Kynurenine Pathway, and Fatty Acids in Plasma Samples of MetS Patients with No Cognitive Impairment (MetS, *N* = 25) and with Mild Cognitive Impairment (MetS-MCI, *N* = 70)^a^

	MetS		MetS-MCI	
	median	IQR	median	IQR
**amino acids (μmol/dL)**				
alanine	50.55	40.90–67.42	48.85	41.03–62.62
glycine	31.66	30.08–34.72	34.38	30.11–39.83
valine	21.89	19.74–25.18	19.55	16.40–24.80
leucine	14.91	14.00–18.35	14.85	13.74–18.11
proline	19.31	15.45–21.17	18.12	14.66–21.03
serine	13.00	10.22–19.17	12.59	9.53–15.63
threonine	5.29	3.86–8.21	4.14	3.22–5.60
phenylalanine	29.82	26.10–34.88	26.19	16.63–30.85
ornithine (arginine)	22.66	20.20–27.26	24.79	19.34–29.17
asparagine	31.37	24.39–39.98	34.60	27.39–39.55
lysine*	26.25	21.99–30.65	22.34	19.95–26.05
histidine	13.32	11.36–16.99	13.65	10.41–16.79
tyrosine	6.50	5.68–7.44	6.30	5.68–7.45
AAA	36.51	19.96–43.46	29.69	19.65–38.83
BCAA	37.47	32.64–50.52	38.63	30.33–49.05
**tryptophan metabolites**				
tryptophan (μmol/dL)	3.36	2.66–3.58	2.84	2.51–3.36
kynurenic acid (nmol/dL)*	2.64	2.01–3.60	3.49	2.64–4.76
kynurenine (nmol/dL)*	88.95	85.39–98.46	104.08	87.34–143.94
picolinic acid (nmol/dL)	0.97	0.81–1.30	0.97	0.81–1.14
quinolinic acid (nmol/dL)	20.46	15.68–34.35	24.47	17.53–44.16
serotonin (nmol/dL)	14.36	11.46–20.09	16.00	12.31–22.02
KTR**	0.0274	0.0253–0.0326	0.0374	0.0299–0.0492
**total fatty acids (μmol/dL)**				
C12:0	5.62	5.59–5.64	6.14	5.29–7.73
C16:0	277.16	202.40–358.71	270.55	210.68–323.56
C16:1 [Z-9]	45.16	35.30–63.87	39.82	30.36–64.47
C18:0	85.27	75.11–106.92	84.06	70.97–105.16
C18:1 [Z-9]	209.67	166.80–341.36	230.58	180.74–307.60
C18:2[Z,Z-9,12]	287.29	216.87–348.97	284.67	212.01–350.46
C14:0	17.38	11.95–26.89	16.51	12.53–24.71
C20:3 [all-Z-8,11,14]**	23.77	23.19–29.10	20.85	18.79–23.67
C20:4 [all-Z-5,8,11,14]	100.18	75.72–103.89	80.81	66.60–91.88
C22:6 [ all-Z-4,7,10,13,16,19]	40.93	35.55–45.59	40.35	34.05–47.52
total FA	1,135.41	855.17–1,354.10	1,120.42	906.09–1,406.77
PUFA	450.59	332.89–502.80	447.79	338.61–517.79
MUFA	264.23	193.11–416.36	286.45	215.73–372.06

a AAA: aromatic amino acids, BCAA: branched-chain amino acids, PUFA: polyunsaturated fatty acids, MUFA: monounsaturated fatty acids; * *p*-value <0.05, ** *p*-value <0.01, and *** *p*-value <0.001 (Mann–Whitney *U* test).

**Figure 2:**
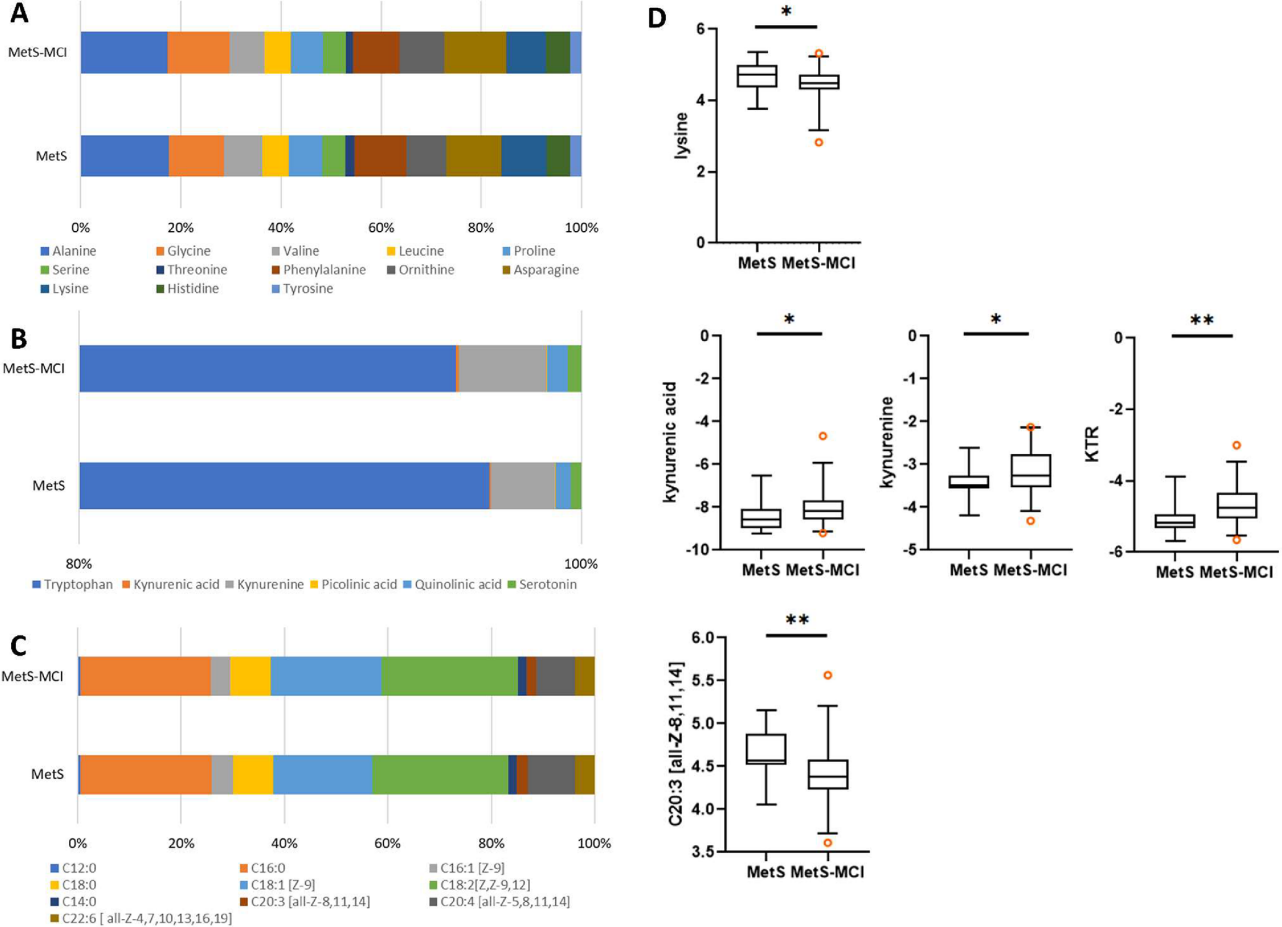
Percent compositions of (A) amino acids, (B) tryptophan metabolites, and (C) fatty acids in plasma samples of MetS and MetS-MCI. (D) Box plots of metabolites exhibiting significant different concentrations in MetS vs MetS-MCI (Mann–Whitney *U* test). *p-value <0.05 and **p-value <0.01.

### Multivariate Analysis of All the Metabolites

We explored metabolic variation between the sample groups from the combined metabolite data set, also including relevant ratios and sums of metabolites, using multivariate analysis. Results from the unsupervised principal component analysis (PCA) of the common metabolites showed no distinct variation between the sample groups (Figure S1). By using the OPLS-DA, the metabolite profiles of MetS-MCI and MetS were slightly separated. The OPLS-DA model explained 28.8% of the group variance (*R* ^2^ *Y* = 0.288) and 44.9% of the metabolite variance (*R* ^2^ *X* = 0.449) but exhibited low predictive power (*Q* ^2^ = −0.165), indicating that the metabolic differences between groups were subtle and the model lacked discriminative robustness (Figure S2A). However, there were five discriminant metabolites with VIP score >1.5, which were kynurenic acid, quinolinic acid, serotonin, aromatic amino acids (AAA), and KTR (Figure S2B). MetS-MCI had higher median concentrations of kynurenic acid, quinolinic acid, serotonin, and KTR but a lower median concentration of AAA compared with those in MetS (Table 3).

### Higher KTR Was Linked to an Increased Likelihood of MCI

In total, there were eight significant candidates identified from the univariate and multivariate analyses: lysine, kynurenic acid, kynurenine, KTR, C20:3 [all-Z-8,11,14], AAA, quinolinic acid, and serotonin. We investigated associations between the eight significant candidates with MCI using binary logistic regression models adjusted for age, gender, and BMI. As shown in Table 4, MCI was positively associated with KTR (*B* = 1.550, *p*-value = 0.014) but negatively associated with C20:3 [all-Z-8,11,14] (*B* = −1.659, *p*-value 0.022) and lysine (*B* = −1.496, *p*-value 0.034) even after the adjustments. In contrast, age, gender, and BMI showed no association with MCI. The natural log-transformed odds ratios of the eight significant metabolites, adjusted for age, BMI, and gender, are shown in Figure 3. KTR resulted in the highest log-odds ratio (1.55, *p*-value = 0.01), indicating that higher KTR was significantly associated with increased odds of developing MCI. A one-unit increase in KTR yields a change in probability of developing MCI by 371%. In contrast, higher levels of C20:3 [all-Z-8,11,14] and lysine were significantly associated with reduced odds of developing MCI (C20:3 [all-Z-8,11,14]: log-odds = −1.66, *p*-value = 0.02, lysine: log-odds = −1.50, *p*-value = 0.03). One-unit increases in C20:3 [all-Z-8,11,14] and lysine decrease the chances of developing MCI by 81 and 78%, respectively.

**Table 4:** Associations between Eight Significant Metabolites and MCI Using Binary Logistic Regression Models Adjusted for Age, Gender, and BMI^a^

	age		gender		BMI		metabolites	
	*B*	*p*-value	*B*	*p*-value	*B*	*p*-value	*B*	*p*-value
KTR	0.026	0.514	–0.344	0.499	–0.047	0.275	**1.550**	**0.014**
C20:3 [all-Z-8,11,14]	0.069	0.068	–0.241	0.632	–0.015	0.723	**–1.659**	**0.022**
kynurenic acid	0.045	0.235	–0.474	0.352	–0.038	0.383	0.744	0.087
kynurenine	0.042	0.282	–0.467	0.358	–0.031	0.450	1.201	0.050
lysine	0.071	0.051	–0.394	0.434	–0.006	0.893	**–1.496**	**0.034**
quinolinic acid	0.050	0.187	–0.303	0.538	–0.032	0.457	0.435	0.194
serotonin	0.070	0.062	–0.184	0.712	–0.012	0.763	0.262	0.402
AAA	0.073	0.051	–0.181	0.714	–0.007	0.867	–0.355	0.210

a *B*: beta coefficient; AAA, aromatic amino acids; and MCI: mild cognitive impairment.

**Figure 3:**
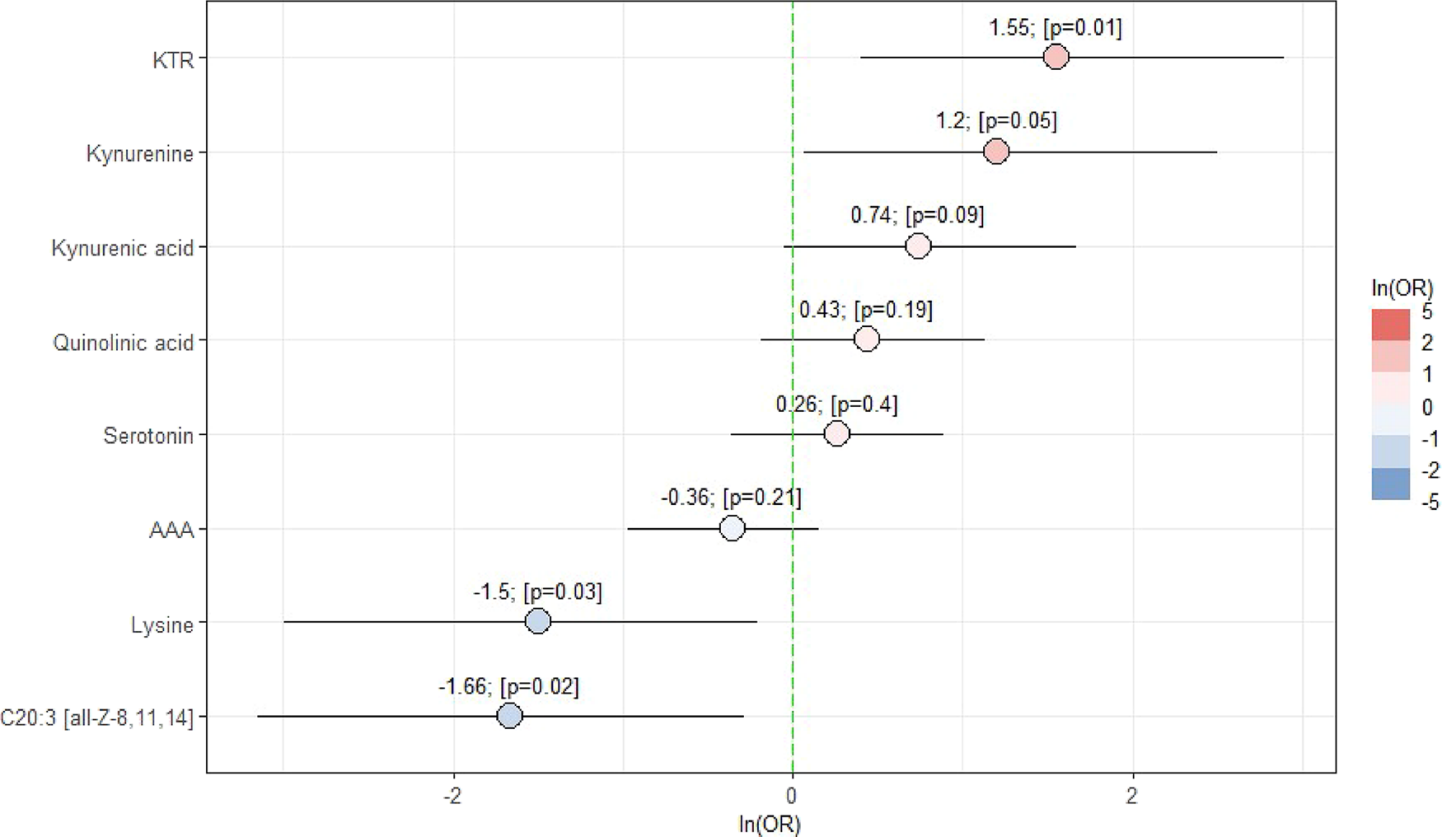
Natural log-transformed odds ratios (ln­(OR)) and 95% confidence intervals for the significant metabolites. *P*-value (*p*).

Spearman correlation analysis of the three significant metabolites with clinical parameters is shown in Table S4. Similarly, KTR exhibited the strongest negative correlation with MoCA scores (*R* = −0.38, *p*-value <0.001), while C20:3 [all-Z-8,11,14] and lysine were positively correlated with MoCA scores (*R* ∼ 0.2, *p*-value <0.05). Other clinical parameters were not correlated with the MoCA score except for insulin (*R* = 0.25, *p*-value <0.05). KTR and C20:3 [all-Z-8,11,14] were positively correlated with triglycerides and VLDL but were negatively correlated with HDL. Only KTR showed a positive correlation with FGF21 (*R* = 0.40, *p*-value <0.001).

## Discussion

In the present study, we validated GC-TOFMS and LC-TQMS methods and quantified three classes of metabolites—amino acids, fatty acids, and tryptophan metabolites—in plasma samples of a treated MetS cohort (*N* = 95). Using the education-adjusted MoCA score cutoff <23, the cohort was classified into MetS (*N* = 25) and MetS-MCI (*N* = 70) groups. There has been an inconsistency with the MoCA cutoff score selection in previous studies.^36,38^ The cutoff <26 was initially recommended,^39^ but later in 2018, a systematic review study conducting meta-analysis suggested a cutoff score of 23 to avoid false positives pertaining to the elderly or low educational background population.^38^ We chose to follow the latter recommendation due to the cohort’s low educational background.

Among the three classes of metabolites quantified in this study, tryptophan metabolites in the kynurenine pathway were apparently altered in MetS-MCI patients, showing a greater concentration of kynurenine. Kynurenine is the first breakdown product of tryptophan in the kynurenine pathway activated by indoleamine 2,3-dioxygenase (IDO) and tryptophan 2,3-dioxygenase (TDO) enzymes. Because the IDO-mediated activation by inflammatory factors elevates kynurenine production,^20^ KTR has been used as an inflammatory marker or a measure of IDO activity. The associations between KTR and inflammatory markers have been reported previously in various diseases.^31,40–42^ Regarding T2D, it has been established that KTR is linked to T2D pathogenesis through insulin resistance—a component of MetS—which is partly driven by chronic inflammation.^20^ KTR values in healthy controls could vary across populations based on various factors, but the levels of KTR were often elevated in people with MetS, T2D, and obesity.^43–46^ An exception was seen in a Norwegian cohort where plasma KTR of T2D and control groups were not significantly different, ranging from 0.0235 to 0.0237.^47^ When compared to previous cohort studies in MetS population, the median KTR of 0.0274 observed in Thai MetS patients measured in this study was ∼1.8 times lower than those reported in Brazilian (∼0.048)^43^ and Austrian MetS (0.0506) cohorts.^44^ The differences could be attributed to many factors such as age,^48,49^ differences in MetS components, or ethnicity which broadly encompasses genetics, food, lifestyle, etc.

Although the precise pathologies of AD, a severe progression of MCI, are debatable, neuroinflammation is regarded as a component contributing to the disease pathology and has been reported to be associated with amyloid Positron Emission Tomography in patients with MCI.^50^ In the MetS condition, chronic inflammation can lead to neuroinflammation due to the crossing of proinflammatory cytokines through the blood–brain barrier.^51^ Previous population-based studies showed that people with MetS and high inflammation levels, assessed by C-reactive protein, were associated with a form of cognitive impairment.^2,52^ A similar finding was reported in a cohort study of T2D using a different panel of inflammation biomarkers (IL-1β and NFκBp65) and cognitive screening tests.^18^ In terms of metabolites, results from a cohort study of elderly individuals without cognition impairment and major health issues showed associations between KTR and neurofilament light chain as well as amyloid-β in blood,^53^ suggesting a strong link between KTR and neuroinflammation.

The association between KTR and cognitive performance has also been previously demonstrated, though not in patients with MetS. A cohort study of the Norwegian elderly population showed an inverse relationship between KTR and cognitive performance after accounting for confounding factors.^54^ Another study reported that almost half of cognitively impaired participants were T2D and had higher plasma KTR compared to cognitively normal participants.^55^ Our finding regarding the association between KTR and MCI, after adjusting for confounders, highlights the prominent neuroinflammation, beyond normal aging, in MetS patients, even under ongoing clinical treatment. It suggests that systemic inflammation plays a role in the development of cognitive impairment. While clinical screening tests for cognitive impairment can be challenging when patients score in the borderline range, this potential fluid biomarker could offer additional information to complement existing clinical evidence.

Fatty acids contribute to cognitive function in several ways, including neurotransmission, maintaining cell integrity, and regulating inflammation.^26,56^ With regard to neurodegenerative diseases, metabolite profiling of MCI or AD commonly revealed decreased levels of plasma or serum fatty acids and more pronounced differences in control vs AD.^26^ In a separate cohort study of individuals with T2D, some fatty acids demonstrated inverse relationships between plasma concentration and total MoCA scores.^18^ Among fatty acids, circulating unsaturated fatty acid concentrations were found to be disturbed in relation to cognitive decline and AD progression.^25^ ^,^ ^26^ In this study, we observed a lower abundance of total fatty acids in MetS-MCI, but this was not statistically significant. In contrast to other studies, we found no significant difference between concentrations of C22:6 [all-Z-4,7,10,13,16,19] or DHA, known as a neuroprotective compound, in MetS vs MetS-MCI. Among the detected fatty acids, only the lower abundance of C20:3 [all-Z-8,11,14] or eicosatrienoic acid in MetS-MCI reached statistical significance. Our measured C20:3 [all-Z-8,11,14] concentrations fall within the same range as those reported previously.^57^ But the previous study reported no significant difference in concentrations between healthy control (19.25 μmol/dL) and MCI (21.93 μmol/dL) groups without MetS. In contrast, in a coronary artery disease cohort, serum C20:3 [all-Z-8,11,14] concentration was significantly lower in coronary artery disease patients with MCI.^58^ Although the relationship between C20:3 [all-Z-8,11,14] and cognitive function is unclear based on population-based studies, metabolized products of C20:3 [all-Z-8,11,14] mediated by cyclooxygenase and 15-lipoxygenase exhibited various biological activities such as anti-inflammation and anticancer.^59^ This suggests that the higher concentration of C20:3 [all-Z-8,11,14] may lead to the production of anti-inflammatory breakdown products, potentially exerting a beneficial effect on neuroinflammation, as reflected by lower KTR levels in MetS patients without MCI.

The higher lysine concentration in MetS patients without MCI than that in MetS patients with MCI implies a positive effect on cognitive performance. In a longitudinal cohort study of the elderly Japanese population, low levels of lysine intake along with phenylalanine, threonine, and alanine were associated with cognitive decline regardless of total protein consumption.^60^ Another study observed a correlation between MMSE score and plasma lysine concentration, and a decreasing trend of lysine concentrations where control > MCI > AD.^7^ In addition, an in vitro study using porcine intestinal cells showed that lysine deficiency induced cell apoptosis, which could be linked to inflammation.^61,62^ Our findings regarding KTR, C20:3, and lysine underscore the close connection of systematic inflammation to cognitive functions in treated MetS patients.

The absence of strong associations between MoCA scores and clinical parameters suggests that the panel of routine clinical tests may not be effective in detecting the onset of cognitive impairment in the treated MetS patients. As such, the proposed metabolites may have greater potential as a risk marker for MCI, complementing the existing cognitive assessments that may be biased by educational background, a predominant confounding factor in populations from developing countries.

Nonetheless, the study had two main limitations: (1) the absence of comprehensive clinical assessments, such as neuroimaging or neurodegeneration markers, to confirm the presence of MCI and (2) the low educational background of the study participants. Future validation studies could benefit from recruiting individuals with a wider range of educational backgrounds and incorporating neuropathological tests. Additionally, employing an integrative analysis that combines multiomics approaches with clinical data will offer a more understanding of the disease pathogenesis.^63^ Based on these findings, future research should focus on uncovering how inflammation (as indicated by KTR) and changes in fatty acid and amino acid metabolism influence brain function in MetS. Studies could explore the biological mechanisms involved and examine these metabolites over time to assess their values as early indicators of MCI or dementia. Stratifying patients by metabolite profiles may also help identify those at greater risk, enabling targeted prevention strategies.

## Conclusions

We applied the validated GC-TOFMS and LC-TQMS methods to quantify amino acids, total fatty acids, and tryptophan metabolites in the kynurenine pathway in the plasma of treated MetS patients with and without MCI assessed by MoCA. Our study provided comprehensive metabolite concentrations, which will be a valuable resource for comparison across studies. We found that MCI was positively associated with KTR after the adjustment for age, gender, and BMI, and, to a lesser extent, was negatively associated with C20:3 [all-Z-8,11,14] and lysine. KTR was significantly associated with increased odds of developing MCI, whereas C20:3 [all-Z-8,11,14] and lysine were associated with decreased odds. The elevated KTR in MetS with MCI suggests a prominent effect of inflammation on cognitive function beyond normal aging and highlights its potential as a risk marker for MCI. This could serve as a valuable complement to existing cognitive assessments, which may be influenced by the educational background.

## Supplementary Material



## Data Availability

All raw data are available in Mendeley Data at 10.17632/2zkgrhfjrx.1.

## Notes

List of targeted compounds; linear ranges, LOQ, and LOD of the target metabolites; quantifications of amino acids and total fatty acids in the NIST plasma SRM 1950; Spearman's correlation analysis of three significant metabolites and clinical parameters; score plot and loading plot from PCA of the combined metabolite data set; and score plot and loading plot from OPLS-DA (PDF)
