# Supplementary material for: Higher Plasma Kynurenine to Tryptophan Correlates with an Increased Incidence of Mild Cognitive Impairment in Treated Metabolic Syndrome Patients
Source: ACS Omega. 2025 Dec 11;10(51):63226–37. doi: 10.1021/acsomega.5c09713 (PMC12756837; doi:10.1021/acsomega.5c09713)

**Supporting Information**

# Higher Plasma Kynurenine to Tryptophan Correlates with Increased Incidence of Mild Cognitive Impairment in Treated Metabolic Syndrome Patients

Narumol Jariyasopit[1,2,3], Tiwat Phochmak[1,3], Siriphan Manocheewa[2,3], Kwanjeera Wanichthanarak[1,2,3], Suphitcha Limjiasahapong[2,3], Nichapa Kleebkomut[1,4], Yongyut Sirivatanauksorn[1,2,3], Vorapan Sirivatanauksorn[2,3,4], Arintaya Phrommintikul[5,6,7], Nipon Chattipakorn[5,6], Siriporn Chattipakorn[5,6,8,*], Sakda Khoomrung[1,2,3,4,9,*]

[1]Siriraj Center of Research Excellence in Metabolomics and Systems Biology, Faculty of Medicine Siriraj Hospital Mahidol University, Bangkok, 10700, Thailand

[2]Siriraj Metabolomics and Phenomics Center, Faculty of Medicine Siriraj Hospital, Mahidol University, Bangkok 10700, Thailand

[3]Thailand Metabolomics Society, Bangkok, Thailand

[4]Department of Biochemistry, Faculty of Medicine Siriraj Hospital Mahidol University, Bangkok, 10700, Thailand

[5]Cardiac Electrophysiology Research and Training Center, Faculty of Medicine, Chiang Mai University, Chiang Mai, 50200, Thailand

[6]Center of Excellence in Cardiac Electrophysiology Research, Faculty of Medicine, Chiang Mai University, Chiang Mai, 50200, Thailand

[7]Department of Internal Medicine, Faculty of Medicine, Chiang Mai University, Chiang Mai, 50200, Thailand

[8]Department of Oral Biology and Diagnostic Sciences, Faculty of Dentistry, Chiang Mai University, Chiang Mai, 50200, Thailand

[9]Center of Excellence for Innovation in Chemistry (PERCH-CIC), Faculty of Science, Mahidol University, Bangkok, Thailand

***Corresponding authors**:

Siriporn C. Chattipakorn, DDS, PhD

Tel:  +66-53-935-329, Fax:  +66-53-935-368  *Email*: siriporn.c@cmu.ac.th;
scchattipakorn@gmail.com
Sakda Khoomrung, PhD
Tel: +66 2419 5506; fax: +66 2411 0155 *E-mail*: sakda.kho@mahidol.edu

**Table S1.** List of targeted compounds measured in this study.

| Target Compound | CAS. No. | Manufacturer | Abbreviation |
|---|---|---|---|
| **Amino acids** | | | |
| L-Alanine | 56-41-7 | Sigma-Aldrich | |
| L-Glycine | 56-40-6 | Sigma-Aldrich | |
| L-Valine | 72-18-4 | Sigma-Aldrich | |
| L-Leucine | 61-90-5 | Sigma-Aldrich | |
| L-Proline | 147-85-3 | Sigma-Aldrich | |
| L-Pyroglutamic acid | 98-79-3 | Sigma-Aldrich | |
| L-Methionine | 63-68-3 | Sigma-Aldrich | |
| L-Serine | 56-45-1 | Sigma-Aldrich | |
| L-Threonine | 72-19-5 | Sigma-Aldrich | |
| L-Phenylalanine | 63-91-2 | Sigma-Aldrich | |
| L-Aspartic acid | 56-84-8 | Sigma-Aldrich | |
| L-Hydroxyproline | 51-35-4 | Sigma-Aldrich | |
| L-Cysteine | 52-90-4 | Sigma-Aldrich | |
| L-Glutamic acid | 56-86-0 | Sigma-Aldrich | |
| DL-Ornithine | 616-07-9 | Sigma-Aldrich | |
| L-Asparagine | 70-47-3 | Sigma-Aldrich | |
| L-Lysine | 56-87-1 | Sigma-Aldrich | |
| L-Histidine | 71-00-1 | Sigma-Aldrich | |
| L-Tyrosine | 60-18-4 | Sigma-Aldrich | |
| L-Tryptophan | 73-22-3 | Sigma-Aldrich | |
| **FAMEs** | | | |
| Methyl dodecanoate | 111-82-0 | Restek | C12:0 |
| Methyl palmitate | 112-39-0 | Restek | C16:0 |
| Methyl palmitoleate | 1120-25-8 | Restek | C16:1 [Z-9] |
| Methyl stearate | 112-61-8 | Restek | C18:0 |
| Methyl oleate | 112-62-9 | Restek | C18:1 [Z-9] |
| Methyl linoleate | 112-63-0 | Restek | C18:2 [Z,Z-9,12] |

| | | | |
|---|---|---|---|
| Methyl linolenate | 301-00-8 | Restek | C18:3 [all-Z-9,12,15] |
| Methyl behenate | 929-77-1 | Restek | C22:0 |
| Methyl myristate | 124-10-7 | Restek | C14:0 |
| Methyl myristoleate | 56219-06-8 | Restek | C14:1 |
| Methyl pentadecanoate | 7132-64-1 | Restek | C15:0 |
| Methyl heptadecanoate | 1731-92-6 | Restek | C17:0 |
| Methyl linoleanate | 16326-32-2 | Restek | C18:3[all-Z-6,9,12] |
| Methyl arachidate | 1120-28-1 | Restek | C20:0 |
| Methyl eicosenoate | 2390-09-2 | Restek | C20:1 [Z-11] |
| Methyl eicosadienoate | 2463-02-7 | Restek | C20:2 [Z,Z-11,14] |
| Methyl eicosatrienoate | 21061-10-9 | Restek | C20:3 [all-Z-8,11,14] |
| Methyl arachidonate | 2566-89-4 | Restek | C20:4 [all-Z-5,8,11,14] |
| Methyl cis 5,8,11,14,17-eicosapentaenoate | 2734-47-6 | Restek | C20:5 [all-Z-5,8,11,14,17] |
| Methyl erucate | 1120-34-9 | Restek | C22:1 [Z-13] |
| Methyl cis-7,10,13,16,19-Docosapentaenoate | 108698-02-8 | Restek | C22:5 [all-Z-,7,10,13,16,19] |
| Methyl docosahexaenoate | 2566-90-7 | Restek | C22:6 [ all-Z-4,7,10,13,16,19] |
| Methyl ligoncerate | 2442-49-1 | Restek | C24:0 |
| Methyl nervonate | 2733-88-2 | Restek | C24:1 [Z-15] |
| Methyl hexacosanoate | 5802-82-4 | Restek | C26:0 |
| **Metabolites in kynurenine pathway** | | | |
| anthranilic acid | 118-92-3 | Sigma-Aldrich | |
| cinnabarinic acid | 606-59-7 | Sigma-Aldrich | |
| kynurenic acid | 492-27-3 | Sigma-Aldrich | |
| kynurenine | 343-65-7 | Sigma-Aldrich | |
| picolinic acid | 98-98-6 | Sigma-Aldrich | |

| | | | |
|---|---|---|---|
| quinolinic acid | 89-00-9 | Sigma-Aldrich | |
| tryptophan | 73-22-3 | Sigma-Aldrich | |
| xanthurenic acid | 59-00-7 | Sigma-Aldrich | |
| 3-hydroxyanthranilic acid | 548-93-6 | Sigma-Aldrich | |
| 3-hydroxykynurenine | 484-78-6 | Sigma-Aldrich | |
| serotonin | 50-67-9 | Sigma-Aldrich | |

**Table S2** Linear ranges, limits of quantitation (LOQ), and limits of detection (LOD) of the target metabolites.

| Compound | Linear range (ng/µL) | LOQ (ng/µL) | LOD (ng/µL) |
|---|---|---|---|
| **Amino acids** | | | |
| Alanine | 1 – 40 | 1 | 0.01 |
| Glycine | 1 – 16 | 1 | 0.01 |
| Valine | 1 – 40 | 1 | 0.01 |
| Leucine | 1 – 40 | 1 | 0.01 |
| Isoleucine | 1 – 40 | 1 | 0.01 |
| Proline | 1 – 40 | 1 | 0.01 |
| Methionine | 1 – 40 | 1 | 0.01 |
| Serine | 0.1 – 40 | 0.1 | 0.10 |
| Threonine | 0.1 – 40 | 0.1 | 0.01 |
| Phenylalanine | 2 – 40 | 2 | 0.02 |
| Aspartic acid | 1 – 24 | 1 | 0.01 |
| Hydroxyproline | 1 – 40 | 1 | 0.01 |
| Cysteine | 0.1 – 40 | 0.1 | 0.02 |
| Glutamic acid | 1 – 40 | 1 | 0.01 |
| Ornithine | 2 – 40 | 2 | 0.10 |
| Asparagine | 1 – 40 | 1 | 0.01 |
| Lysine | 1 – 40 | 1 | 0.01 |
| Histidine | 1 – 40 | 1 | 1.00 |
| Tyrosine | 1 – 40 | 1 | 0.01 |
| Tryptophan | 1 – 40 | 1 | 0.10 |
| | | | |
| **Tryptophan metabolites** | **ng/mL** | **ng/mL** | **ng/mL** |
| Tryptophan | 5000 – 12000 | 0.72 | 0.22 |
| Kynurenine | 137.12 – 1387.12 | 0.30 | 0.09 |
| Kynurenic acid | 5.74 – 130.74 | 1.49 | 0.45 |
| Anthanilic acid | 1 – 126 | 1.69 | 0.51 |
| 3-OH-anthranilic acid | 1.98 – 63.50 | 1.29 | 0.39 |
| 3-OH-kynurenine | 1 – 63.50 | 1.50 | 0.45 |
| Picolinic acid | 0.88 – 63.38 | 0.28 | 0.08 |
| Quinolinic acid | 40.18 – 540.18 | 2.26 | 0.68 |
| Serotonin | 18.82 – 268.82 | 2.18 | 0.65 |
| Xanthurenic acid | 1 – 63.50 | 0.34 | 0.10 |
| | | | |
| **Fatty acid methyl esters** | **ng/µL** | **ng/µL** | **ng/µL** |
| C12:0 | 0.5-20.0 | 0.09 | 0.26 |
| C16:0 | 7.5-90.0 | 0.15 | 0.47 |
| C16:1(Z-9) | 1.0-40.0 | 0.09 | 0.27 |
| C18:0 | 1.0-40.0 | 0.15 | 0.45 |
| C18:3[all-Z-9,12,15] | 0.5-20.0 | 0.17 | 0.50 |
| C18:1 [Z-9] | 2.0-80.0 | 0.15 | 0.46 |

| | | | |
|---|---|---|---|
| C18:2 [Z,Z-9,12] | 5.0-100.0 | 0.12 | 0.38 |
| C22:0 | 1.0-10.0 | 0.16 | 0.49 |
| C14:0 | 0.5-20.0 | 0.12 | 0.37 |
| C14:1 | 0.5-10.0 | 0.16 | 0.47 |
| C15:0 | 0.25-10.0 | 0.08 | 0.25 |
| C17:0 | 0.5-20.0 | 0.11 | 0.34 |
| C18:3[all-Z-6,9,12] | 2.5-20.0 | 0.17 | 0.50 |
| C20:0 | 0.25-10.0 | 0.07 | 0.21 |
| C20:1 [Z-11] | 1.0-20.0 | 0.10 | 0.29 |
| C20:2 [Z,Z-11,14] | 1.0-20.0 | 0.22 | 0.68 |
| C20:3 [all-Z-8,11,14] | 1.0-20.0 | 0.24 | 0.72 |
| C20:4 [all-Z-5,8,11,14] | 2.5-40.0 | 0.26 | 0.78 |
| C20:5 [all-Z-5,8,11,14,17] | 1.0-20.0 | 0.23 | 0.69 |
| C22:1 [Z-13] | 1.0-20.0 | 0.29 | 0.88 |
| C22:5 [all-Z-,7,10,13,16,19] | 1.0-20.0 | 0.27 | 0.82 |
| C22:6 [ all-Z-4,7,10,13,16,19] | 1.0-20.0 | 0.30 | 0.92 |
| C24:0 | 0.5-10.0 | 0.15 | 0.46 |
| C24:1 [Z-15] | 1.0-20.0 | 0.26 | 0.79 |
| C26:0 | 0.5-20.0 | 0.10 | 0.31 |
| | | | |

**Table S3.** Quantifications of amino acids and total fatty acids in the NIST plasma SRM 1950.

| Compounds | Certified Value (μmol/L) | This study (N=3, μmol/L) | % Error |
|---|---|---|---|
| **Amino acids** | | | |
| alanine | 300 ± 26 | 397 ± 6 | 32% |
| glycine | 245 ± 16 | 223 ± 9 | 9% |
| leucine | 100.4 ± 6.3 | 117 ± 13 | 17% |
| histidine | 72.6 ± 3.6 | 119 ± 21 | 64% |
| isoleucine | 55.5 ± 3.4 | 53 ± 9 | 4% |
| Lysine | 140 ± 14 | 151 ± 9 | 8% |
| methionine | 22.3 ± 1.8 | - | 100% |
| proline | 177 ± 9 | 188 ± 15 | 6% |
| Serine | 95.9 ± 4.3 | - | 100% |
| threonine | 119.5 ± 6.1 | 127 ± 8 | 6% |
| tyrosine | 57.3 ± 3.0 | 56 ± 5 | 2% |
| valine | 182.2 ± 10.4 | 214 ± 16 | 17% |
| **Fatty acids** | | | |
| C12:0 | 9.47 ± 0.57 | 6.66 ± 0.94 | 30% |
| C16:0 | 2,364 ± 77 | 1,687 ± 12 | 29% |
| C16:1 [Z-9] | 215 ± 26 | 166 ± 2 | 23% |
| C18:0 | 644 ± 41 | 649 ± 2 | 1% |
| C18:3[all-Z-9,12,15] | 54.6 ± 3.6 | 42 ± 3 | 23% |
| C18:1 [Z-9] | 1,614 ± 154 | 1,130 ± 6 | 30% |
| C18:2 [Z,Z-9,12] | 2,838 ± 143 | 2,174 ± 4 | 23% |
| C22:0 | 47.8 ± 4.6 | 38 ± 1 | 2% |

**Table S4.** Spearman correlation analysis of three significant metabolites (KTR, C20:3, and lysine) and clinical parameters.

| **All** | KTR | C20:3 | Lysine | MoCA_score | FGF21 | Glucose | Insulin | Triglycerides | HDL | LDL | VLDL | Cholesterol | HbA1C |
|---|---|---|---|---|---|---|---|---|---|---|---|---|---|
| KTR | 1.00 | -0.02 | -0.21* | -0.38*** | 0.40*** | 0.09 | 0.08 | 0.29** | -0.21* | -0.06 | 0.22* | -0.07 | 0.16 |
| C20:3 [all-Z-8,11,14] | | 1.00 | 0.04 | 0.23* | 0.10 | 0.04 | 0.15 | 0.24* | -0.26* | 0.19 | 0.22* | 0.17 | 0.00 |
| Lysine | | | 1.00 | 0.27** | -0.09 | -0.06 | 0.15 | 0.09 | -0.07 | -0.03 | 0.08 | -0.13 | -0.02 |
| MoCA_score | | | | 1.00 | -0.19 | -0.19 | 0.25* | -0.06 | -0.02 | -0.05 | -0.09 | -0.14 | -0.15 |
| FGF21 | | | | | 1.00 | 0.10 | -0.09 | 0.34** | -0.20 | 0.00 | 0.28** | -0.02 | 0.19 |
| Glucose | | | | | | 1.00 | 0.10 | 0.28** | -0.27 ** | 0.00 | 0.26* | -0.03 | 0.42*** |
| Insulin | | | | | | | 1.00 | 0.15 | -0.23 * | -0.06 | 0.11 | -0.09 | 0.06 |
| Triglycerides | | | | | | | | 1.00 | -0.47*** | 0.28** | 0.90*** | 0.22 * | 0.22* |
| HDL | | | | | | | | | 1.00 | 0.10 | -0.36*** | 0.25 * | -0.30 ** |
| LDL | | | | | | | | | | 1.00 | 0.30** | 0.86*** | -0.06 |
| VLDL | | | | | | | | | | | 1.00 | 0.29 ** | 0.14 |
| Chloresterol | | | | | | | | | | | | 1.00 | -0.08 |
| HbA1C | | | | | | | | | | | | | 1.00 |

Positive Negative

**Figure S1.** A. score plot and B. loading plot from PCA of the combined metabolite dataset.

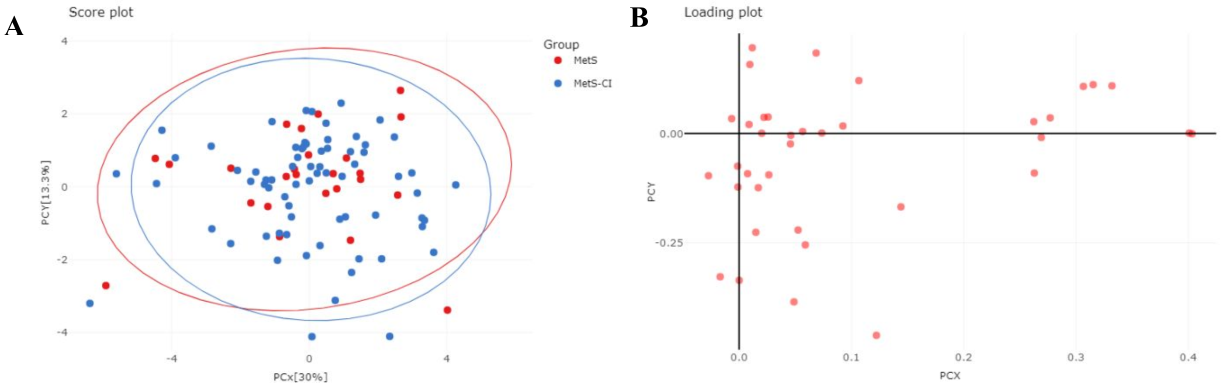

**Figure S2.** A. Score plot and B ($R^2X$ (cum) 0.449, $Q^2$ (cum) -0.165). loading plot from OPLS-DA. The combined metabolite dataset of metabolic syndrome patients with normal cognitive functions (MetS) and with mild cognitive impairment (MetS-MCI) was used in this analysis. Metabolites shown in the loading plot are those with VIP score > 1.5. (AAA: aromatic amino acids)

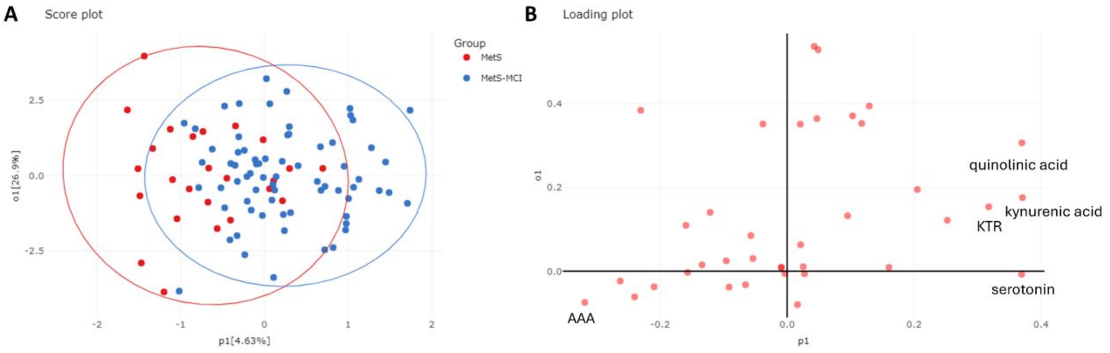

Supplement: Supplementary file 1 [file ao5c09713_si_001.pdf]
